# Radiological differences between chronic thromboembolic pulmonary disease (CTEPD) and chronic thromboembolic pulmonary hypertension (CTEPH)

**DOI:** 10.1007/s00330-020-07556-4

**Published:** 2021-01-28

**Authors:** Carmine Capone, Adele Valentini, Silvia Lina Spinillo, Catherine Klersy, Anna Celentano, Maurizio Pin, Cristian Monterosso, Roberto Dore, Emilio Maria Bassi, Michela Zacchino, Giuseppe Rodolico, Angelo Guido Corsico, Lorenzo Preda, Stefano Ghio, Andrea Maria D’Armini

**Affiliations:** 1 Department of Clinical, Surgical, Pediatric and Diagnostic Sciences, Faculty of Medicine and Surgery, University of Pavia, Pavia, Italy; 2 Diagnostic Imaging Service, ICS Maugeri, Via Salvatore Maugeri 10, 27100 Pavia, PV Italy; 3 Radiology Department, Fondazione IRCCS Policlinico San Matteo, Pavia, Italy; 4 Clinic Epidemiology and Biometry Unit, Fondazione IRCCS Policlinico San Matteo, Pavia, Italy; 5 Unit of Cardio-Thoracic Surgery, Maria Cecilia Hospital SpA, Cotignola, Emilia-Romagna Italy; 6 Division of Cardiac Surgery, Fondazione IRCCS Policlinico San Matteo, Pavia, Italy; 7 Radiology Unit, Gruppo San Donato, Cinical Institute Citta di Pavia, Pavia, Italy; 8 Respiratory Diseases Unit, Department of Medical Sciences and Infectious Diseases, ERN Lung, Fondazione IRCCS Policlinico San Matteo, Pavia, Italy; 9 Department of Internal Medicine and Therapeutics, Faculty of Medicine and Surgery, University of Pavia, Pavia, Italy; 10 Division of Cardiology, Fondazione IRCCS Policlinico San Matteo, Pavia, Italy; 11 Cardiac Surgery and Pulmonary Hypertension Unit, Fondazione IRCCS Policlinico San Matteo, Pavia, Italy

**Keywords:** Pulmonary hypertension, Pulmonary embolism, Dual-energy computed tomography

## Abstract

**Objectives:**

The aim of this study was to describe the radiological features of chronic thromboembolic pulmonary disease (CTEPD), not yet systematically described in the literature. Furthermore, we compared vascular scores between CTEPD and chronic thromboembolic pulmonary hypertension (CTEPH) patients, trying to explain why pulmonary hypertension does not develop at rest in CTEPD patients.

**Methods:**

Eighty-five patients (40 CTEPD, 45 CTEPH) referred to our centre for pulmonary endarterectomy underwent dual-energy computed tomography pulmonary angiography (DE-CTPA) with iodine perfusion maps; other 6 CTEPD patients underwent single-source CTPA. CT scans were reviewed independently by an experienced cardiothoracic radiologist and a radiology resident to evaluate scores of vascular obstruction, hypoperfusion and mosaic attenuation, signs of pulmonary hypertension and other CT features typical of CTEPH.

**Results:**

Vascular obstruction burden was similar in the two groups (*p* = 0.073), but CTEPD patients have a smaller extension of perfusion defects in the iodine map (*p* = 0.009) and a smaller number of these patients had mosaic attenuation (*p* < 0.001) than CTEPH patients, suggesting the absence of microvascular disease. Furthermore, as expected, the two groups were significantly different considering the indirect signs of pulmonary hypertension (*p* < 0.001).

**Conclusions:**

CTEPD and CTEPH patients have significantly different radiological characteristics, in terms of signs of pulmonary hypertension, mosaic attenuation and iodine map perfusion extension. Importantly, our results suggest that the absence of peripheral microvascular disease, even in presence of an important thrombotic burden, might be the reason for the absence of pulmonary hypertension in CTEPD.

**Key Points:**

• *CTEPD and CTEPH patients have significantly different radiological characteristics*.

• *The absence of peripheral microvascular disease might be the reason for the absence of pulmonary hypertension in CTEPD*.

## Introduction

Chronic thromboembolic pulmonary disease (CTEPD) is characterised by the presence of chronic thromboembolic material in the pulmonary arteries without pulmonary hypertension at rest. CTEPD patients represent a small proportion of the patients referred to expert centres, with symptoms and quality of life that may be as poor as those of patients with chronic thromboembolic pulmonary hypertension (CTEPH). Therefore, some of these patients are currently offered the same surgical or interventional treatment as patients with CTEPH [1, 2].

However, there are a number of gaps of evidence regarding this pathological condition.

It is yet unclear why pulmonary hypertension does not develop at rest in such patients; this could be either because the number of occluded segments is insufficient to affect resistance at rest or because no distal vasculopathy has developed as in CTEPH patients. In addition, it is unknown whether exercise limitation is due to exercise-induced pulmonary hypertension, developing in CTEPD due to an increased slope of the pulmonary arterial pressure-flow relationship, or to dead-space ventilation, with increased ventilatory equivalents for carbon dioxide [3, 4]. The natural history of CTEPD is unknown and there is no evidence that CTEPD necessarily evolves to CTEPH [5].

Finally, a relevant gap of evidence in clinical practice is that the radiological features of CTEPD are not systematically described in the literature. Current belief is that angiography, whether computed tomography pulmonary angiography (CTPA) or digital subtraction angiography (DSA), shows in CTEPD patients the typical findings of CTEPH patients [5].

Accordingly, the aim of this study was to compare imaging scores of vascular obstruction and distal perfusion in patients with CTEPD and CTEPH [6–9]. We reasoned that, in the hypothesis that pulmonary hypertension does not develop in CTEPD because the number of occluded segments is insufficient to affect resistance at rest, imaging should demonstrate lesser degree of arterial obstruction in CTEPD. On the contrary, in the hypothesis that pulmonary hypertension does not develop in CTEPD because no distal vasculopathy has developed, imaging could demonstrate better indices of distal perfusion in CTEPD than in CTEPH patients.

## Materials and methods

### Study subjects

Between April 1994 and April 2019, a total number of 936 patients were referred to our centre and underwent pulmonary endarterectomy (PEA); out of these, we retrospectively evaluated all consecutive patients with CTEPD who underwent PEA (*n* = 46). A similar number of consecutive patients (*n* = 45) with CTEPH who underwent PEA in our centre between January 2014 and June 2016, having similar age and sex, was used as the control group, selected from a total number of 97 patients.

The only comorbidity which was considered as an exclusion criterion in both groups was the concomitant presence of parenchymal lung disease, on the basis of ventilation/perfusion scintigraphy data and of the presence of advanced pulmonary emphysema and/or fibrosis at CT (30 patients were excluded only in the CTEPH group).

None of the patients enrolled in our study was referred to our centre for acute pulmonary embolism.

Patients were diagnosed as CTEPH or CTEPD on the basis of all examinations, including right heart catheterisation (RHC) at rest and imaging (CTPA and V/Q scintigraphy), consistently suggested by international guidelines over the study enrollment period [10].

Preoperatively, patients also underwent echocardiographic and respiratory function evaluations.

The respiratory evaluation included spirometry, single-breath transfer factor of the lung for carbon monoxide (TLCO) and arterial blood gas analyses.

The study was approved by the Institutional Review Board of Fondazione IRCCS Policlinico San Matteo (protocol number 20200031903).

### Methods

In CTEPD patients studied before 2009 (*n* = 6), examinations were performed on a 16-slice single-source CT scanner (Somatom Sensation, Siemens Healthineers); all other examinations (both in CTEPD and in CTEPH patients) were performed on a dual-source 64-slice CT scanner (Somatom Definition, Siemens Healthineers).

#### CT protocols

In all patients, two consecutive angiographic scans were performed, in full inspiration.

• Scan parameters for 16-slice CT were as follows: 120 kV with tube current 150 mA; collimation 16 × 0.75; gantry rotation time 50 ms; pitch 0.85. Modulation of the milliamperage was routinely used (Care Dose 4D, Siemens Healthineers).

Pulmonary angiographic acquisition was made with bolus tracking technique, ROI (region of interest) on the main pulmonary artery (threshold 100 HU, scan delay 7 s) and aortic angiographic acquisition with an 8-s delay, with caudo-cranial acquisition for both scans.

We used 100 ml of contrast agent (iomeprol 400 mgI/mL; Iomeron, Bracco), flush at 4 ml/s, followed by 40 ml of normal saline, flush at 4 ml/s.

• Scan parameters for 64-slice dual-source CT were as follows: tube A, 140 kV with tube current 60 mA, FoV diameter 50 cm; tube B, 80 kV with tube current 300 mA, FoV diameter 26 cm; collimation 14 × 1.2 mm; gantry rotation time 30 ms; pitch 0.7. Modulation of the milliamperage was routinely used (Care Dose 4D, Siemens Healthineers).

Pulmonary angiographic acquisition was made with bolus tracking technique, ROI on the main pulmonary artery (threshold 100 HU, scan delay 4 s), and aortic angiographic acquisition with a 4-s delay, with cranio-caudal acquisition for both scans.

We used 65 ml of contrast agent (iomeprol 400 mgI/mL; Iomeron, Bracco), flush at 5 ml/s, followed by 40 ml of normal saline, flush at 5 ml/s.

#### CTPA data reconstruction

From the raw spiral projection data acquired with both tubes of the dual-source CT, “fused” lung and mediastinal images were generated by merging 60% of 140-kV data with 40% of 80-kV data via the process of linear blending [11–13], using medium-soft (D30) convolution kernel for mediastinal images and sharp (B80) convolution kernel for lung images, with 3-mm and 1.5-mm thickness. Reconstructed images were transferred to a commercially available workstation (Leonardo, Siemens Healthineers) running Syngo software (Siemens Healthineers).

From both 80-kV and 140-kV images of the pulmonary angiographic scan, the software generated a dual-energy CT perfusion map, deriving the iodine content of each voxel using a three-material-decomposition algorithm for air, soft tissue and iodine [14]; colour-coded lung PBV images 7 mm in thickness were reconstructed at 14-mm intervals in both the axial and coronal planes using the same dual-energy application software.

From raw data of the single-source scanner, mediastinal and lung images were generated using very smooth (B10) convolution kernel for mediastinal images and ultra-sharp (B80) convolution kernel for lung images, with 1-mm thickness.

#### Image analysis

One cardiothoracic radiologist with more than 10 years of experience and a 5th year radiology resident independently reviewed lung and pulmonary CTPA images and dual-energy CT perfusion maps in a blinded non-consecutive manner, using scores as described by Hoey et al [6].

##### Vascular obstruction

Reviewing pulmonary CTPA images, a modified Qanadli index [15] as described by Hoey et al [6] was used to quantify the degree of arterial obstruction.

A maximal score of 2 was assigned for a patent segmental artery and its first-order subsegmental branches, giving a total possible score of 40 (three branches to both upper lobes, two branches to the middle lobe and lingula, and five branches to both lower lobes). One point was subtracted if there was clear evidence of first-order subsegmental occlusion, a segmental web stenosis or segmental partial occlusion. If two or more of these abnormalities were present, a score of 0 was assigned to that segment. Segmental total occlusion was also assigned a score of 0. Laminated thrombus of the principal or lobar arteries that exceeded 50% of luminal reduction necessitated the subtraction of 1 from the scores for all segmental arterial branches distal to this point. Total vascular obstructive index was calculated by dividing the patient score by 40 and multiplying the result by 100 [15]. Scores for each lobe were expressed as a percentage of normal.

##### Mosaic hyperaemia

Lung images were reviewed. In contrast to the original study [6], we decided to evaluate the ground glass alterations because more easily assessable, referred as “hyperaemic lung parenchyma”.

We assessed the presence of hyperaemic lung parenchyma and the distribution of this alteration (central, peripheric or widespread). Then, a hyperaemic lung parenchyma score was derived by subjective assessment of each lobe for the extent (percentage score at 10% increments; range, 0–100%) of increased attenuation, which was defined as a subjective increase in parenchymal attenuation and containing vessels of larger than expected calibre, corresponding to areas of lung hyperaemia. A total “hyperaemia score” was calculated by applying a weighting factor to each lobar score and adding these together. The weighting factor was based on a 20-segment arterial anatomy model, as described by Qanadli et al [15]: the weighting factors were 3/20 for each upper lobe, 2/20 for the middle lobe and lingula, and 5/20 for each lower lobe.

##### Dual-energy CT perfusion

A dual-energy CT hypoperfusion score was derived by subjective assessment of the iodine map, generated from pulmonary angiographic scan. CTEPD patients who underwent CT on single-source scan were excluded from this evaluation.

Each lobe was scored according to the extent (percentage score at 10% increments; range, 0–100%) of reduced perfusion (areas coloured dark red and black; areas of normal lung are coloured red). Map images were reviewed in axial, coronal and sagittal planes. For lobes not fully included in the dual-energy CT reconstruction field, in patients with thorax diameter exceeding the diameter of tube B FoV, a percentage was taken of the included portion of that lobe. Both readers used their experience to recognise and discount beam-hardening artifacts from undiluted contrast media and cardiac pulsation artifacts. To achieve a total hypoperfusion score, the individual scores for each lobe were weighted and added together in the same way as the mosaic attenuation score was derived.

We also evaluated the frequency of proximal pulmonary occlusion in both groups (considering thrombi obstructing > 50% of the lumen of a principal pulmonary artery).

Other parameters evaluated were the presence of CT indirect signs of pulmonary hypertension, in particular pulmonary artery (PA) diameter (maximum diameter on transversal images), the ratio between PA and aortic diameters (PA/aorta ratio), the ratio between right ventricle (RV) and left ventricle (LV) diameters (RV/LV ratio), the presence of RV hypertrophy (cut-off thickness 4 mm) and interventricular septum bowing, the presence of systemic collateral arteries (bronchial, intercostal and phrenic arteries) and peripheral scars.

### Analysis

Continuous data were described as median and interquartile range (IQR) and categorical data as counts and percent; data were compared with the Mann-Whitney *U* test and the Fisher exact test, respectively. Bonferroni correction was applied for post hoc subgroup comparisons. The Spearman *R* and its 95% confidence interval (95% CI) were computed to assess the association of continuous variables. The Lin’s concordance correlation coefficient and 95% CI were calculated to measure the interobserver agreement in CT readings.

Stata 16 (StataCorp) was used for computations. A two-sided *p* value < 0.05 was considered statistically significant.

## Results

### Patients’ characteristics (Table 1)

The population of this study consisted of 91 patients (48 men and 43 women): 46 CTEPD (28 men and 18 women), 45 CTEPH (20 men and 25 women).

**Table 1:** Baseline characteristics

	CTEPD group (*n* = 46)	CTEPH group (*n* = 45)	*p* value
Sex			
M	28 (61%)	20 (44%)	*p* = 0.144
Age	61 (43–71)	64 (53–69)	*p* = 0.769
BMI	27 (25–30)	26 (23–28)	*p* = 0.088
WHO functional class			
III–IV	21 (46%)	30 (67%)	*p* = 0.058
Disease duration (years)	1.0 (0.0–3.5)	1.0 (1.0–3.0)	*p* = 0.433
PVR (WU)	3.0 (2.4–3.5)	10.6 (6.6–13.2)	*p* < 0.001
mPAP (mmHg)	21 (19–22)	47 (33–53)	*p* < 0.001
CI	2.6 (2.3–3.0)	2.0 (1.7–2.5)	*p* < 0.001
RA pressure (mmHg)	2 (1–5)	6 (3–11)	*p* < 0.001
6MWT (m)	430 (317–490)	297 (228–378)	*p* < 0.001
PaO_2_ (mmHg)	79.8 (70.5–86.8)	68.3 (60.9–76.7)	*p* < 0.001
FEV1 (% pred.)	93 (76–100)	90 (80–104)	*p* = 0.847
FVC (% pred.)	96 (82–102)	100 (88–113)	*p* = 0.100
FEV1/FVC (%)	75 (70–79)	72 (67–77)	*p* = 0.076
TLCO (% pred.)	70 (63–80)	65 (58–76)	*p* = 0.223

*BMI* body mass index, *WHO* World Health Organization, *PVR* pulmonary vascular resistance, *WU* Wood unit, *mPAP* mean pulmonary artery pressure, *CI* cardiac index, *RA* right atrium, *6MWT* 6-min walking test, *PaO*_*2*_ partial pressure of oxygen in arterial blood, *FEV1* forced expiratory volume in 1 s % predicted, *FVC* forced vital capacity % predicted, *TLCO* single-breath carbon monoxide transfer factor % predicted

As expected, patients with CTEPD had a less severe disease considering clinical conditions, right heart hemodynamic profile and functional capacity.

### CTPA findings (Table 2)

**Fig. 1:**
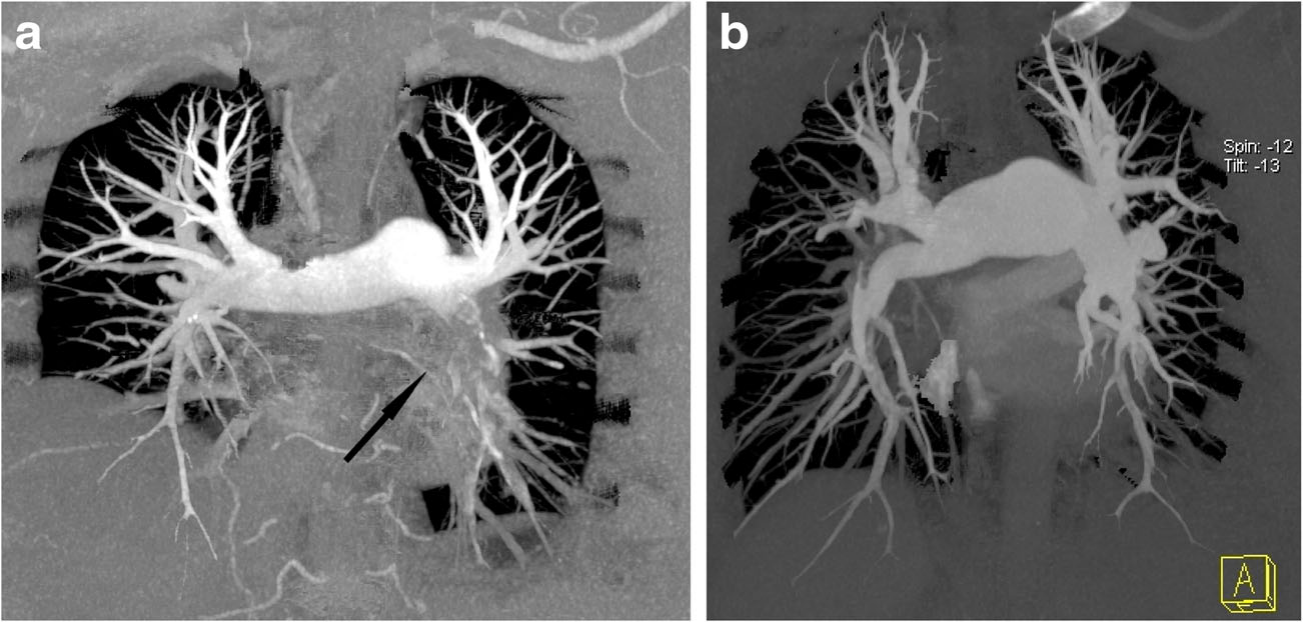
CT pulmonary angiography (coronal plane, MIP reconstructions). **a** CTEPD: proximal occlusion in the left interlobar pulmonary artery (black arrow) and bilateral segmental and subsegmental vascular obstructions. **b** CTEPH: bilateral segmental and subsegmental vascular obstructions

**Fig. 2:**
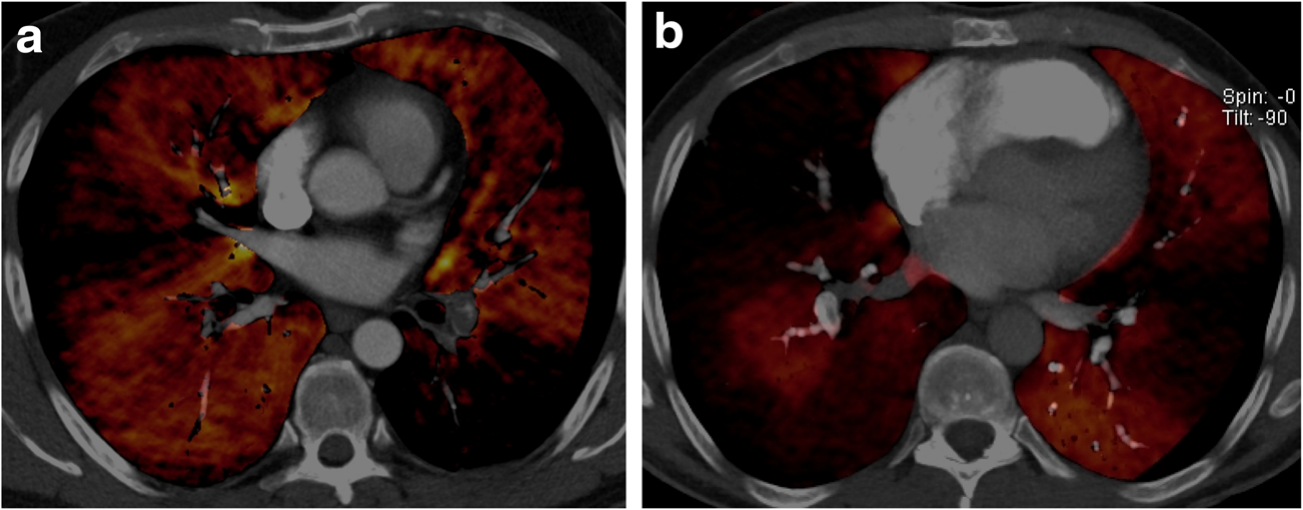
Colour-coded CT iodine map shows a greater extent of perfusion defects (black areas) in CTEPH (**b**) rather than in CTEPD (**a**)

**Fig. 3:**
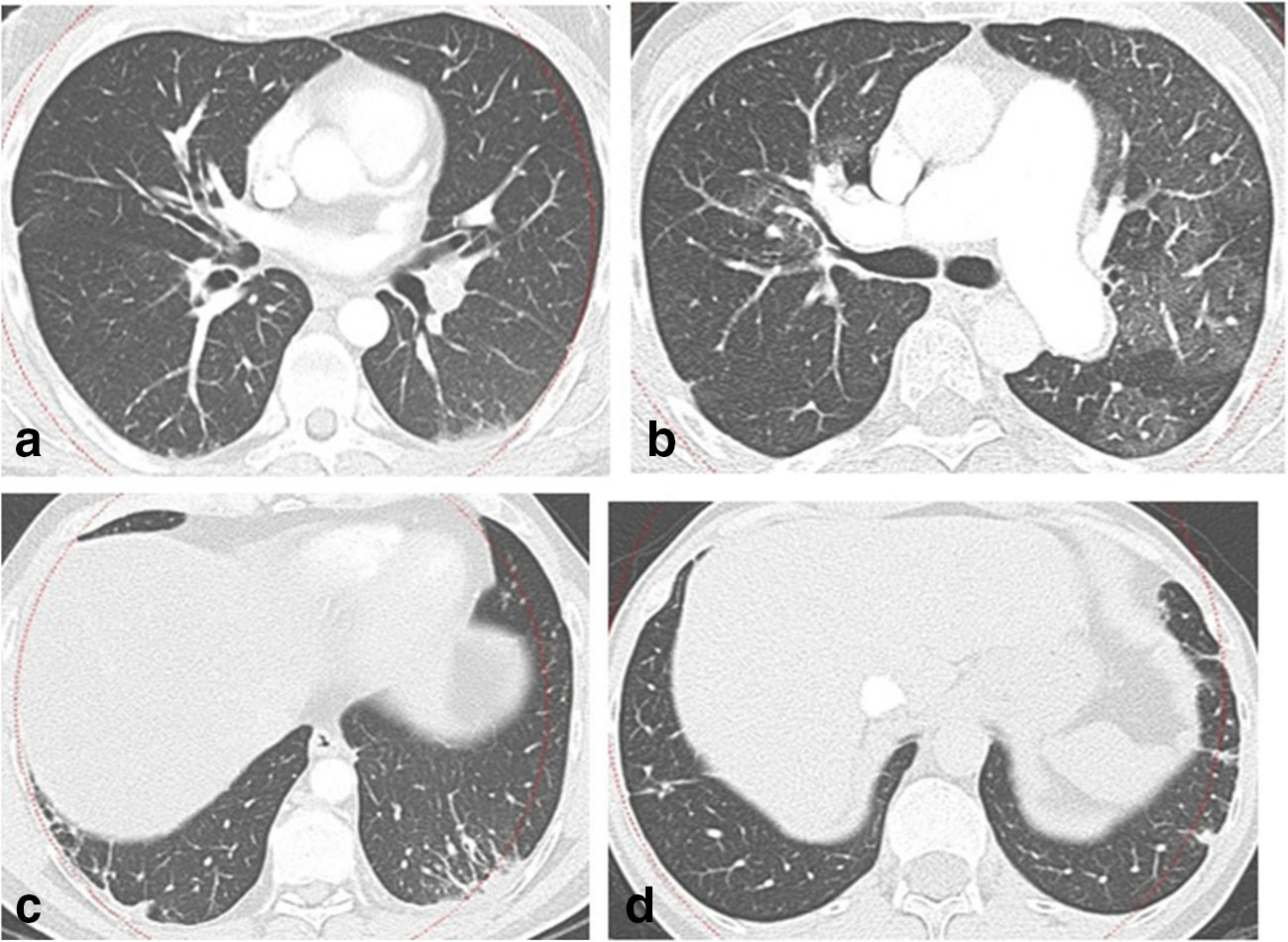
Lung window (b80 kernel reconstruction) CT. Lung hyperaemia is well evident in CTEPH (**b**) with both central and peripheral distribution, whereas is absent in CTEPD (**a**). Peripheral scars are present in both CTEPD (**c**) and CTEPH (**d**) patients

**Fig. 4:**
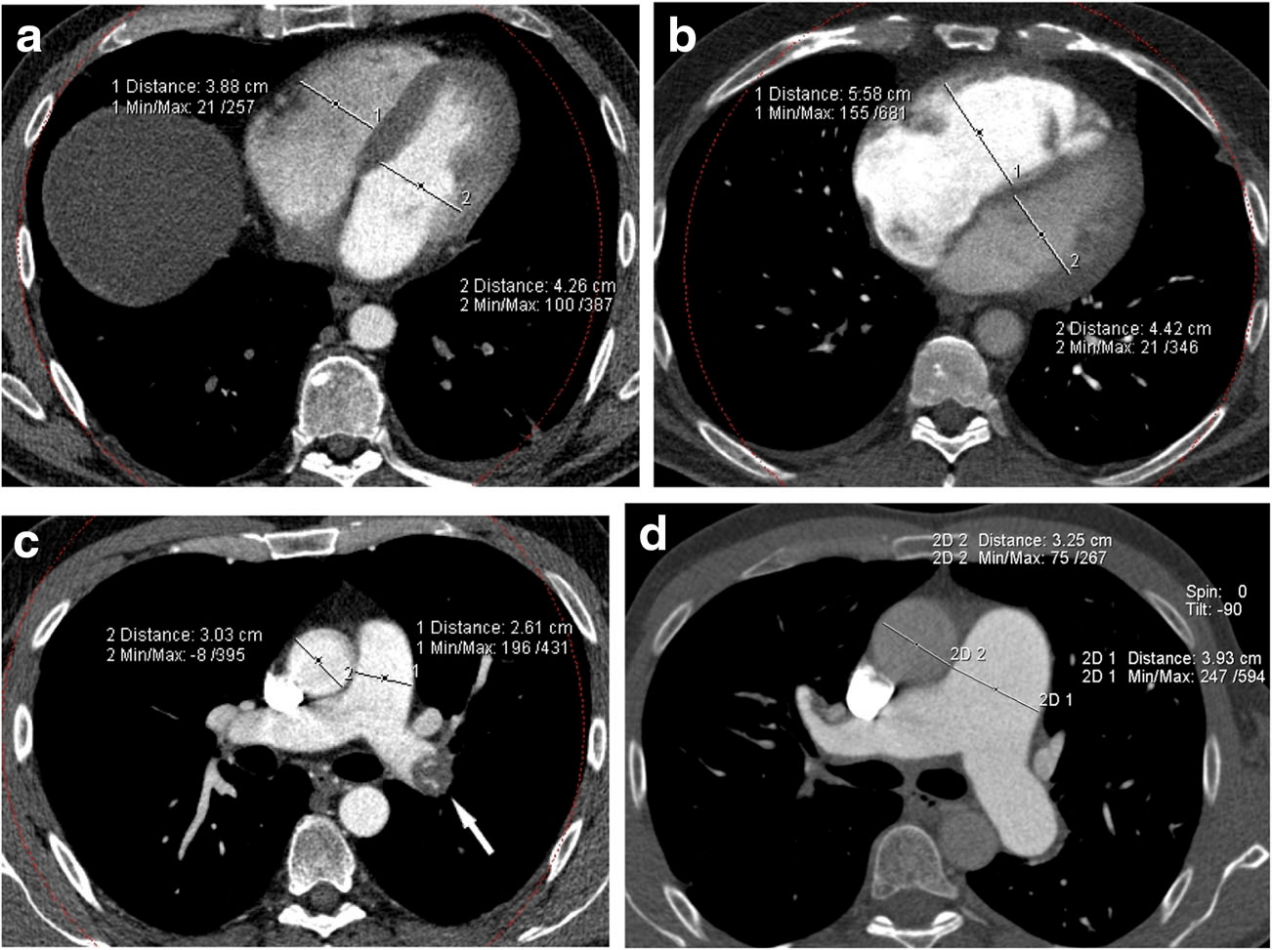
CT signs of pulmonary hypertension. Normal heart diameters in CTEPD (**a**); in CTEPH patients (**b**) right ventricle dilation with septum bowing and heart leftward rotation are present. Important main pulmonary artery dilation in CTEPH (**d**), absent in CTEPD (**c**); proximal left main artery obstruction is evident in (**c**) (white arrow)

**Fig. 5:**
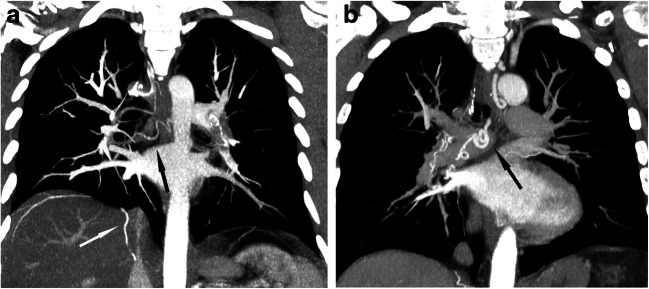
CT angiography (MIP reconstructions) shows hypertrophic bronchial arteries in both CTEPD (**a**) and CTEPH (**b**) patients (black arrows); a hypertrophic phrenic artery is also evident in (**a**) (white arrow)

- Vascular obstruction (Fig. 1)—median vascular obstructive score was 40% in the CTEPD group and 35% in the CTEPH group, with a non-significant difference between the two groups (*p* = 0.073).
- Proximal pulmonary obstruction—proximal obstruction was present in 14 patients (29%) with CTEPD and in 5 patients (11%) with CTEPH (*p* = 0.040).
- Hypoperfusion score (Fig. 2)—median hypoperfusion score was 47% in the CTEPD group and 57% in the CTEPH group, with a significant difference between the two groups (*p* = 0.009).
- Lung hyperaemia (Fig. 3)—mosaic attenuation in lung parenchyma was observed in 8 patients (17%) with CTEPD and in 39 patients (87%) with CTEPH (*p* < 0.001); where present, there was no significant difference neither in its distribution nor in the hyperaemia score (median 20.25% in the CTEPD group, 20% in the CTEPH group; *p* = 0.581).
- Pulmonary hypertension signs (Fig. 4)—a significant difference (*p* < 0.001) between the CTEPD and CTEPH groups was found for all indirect signs of pulmonary hypertension considered (RV hypertrophy, RV/LV diameters ratio, septum bowing, PA diameter and PA/aortic diameters ratio). Modest correlations were observed between PA diameter and both mPAP (*r* = 0.472; *p* < 0.001) and PVR (*r* = 0.477; *p* < 0.001) and between PA/aorta ratio and both mPAP (*r* = 0.429; *p* < 0.001) and PVR (*r* = 0.417; *p* < 0.001).
- Collateral systemic supply (Fig. 5) and peripheral scars (Fig. 3)—hypertrophic bronchial, intercostal and phrenic arteries were found in both CTED and CTEPH patients, without significant differences between the two groups (*p* = 0.476, *p* = 0.268 and *p* = 0.082 respectively), such as peripheral scars (*p* = 1.000).

**Table 2:** CT characteristics

	CTEPD group (*n* = 46)	CTEPH group (*n* = 45)	*p* value
Vascular obstruction score (%)	40 (35–52.5)	35 (30–47.5)	*p* = 0.073
Mosaic hyperaemia	8 (17%)	39 (87%)	*p* < 0.001
Hyperaemia score (%)	20.25 (9.8–26.3)	20 (14.5–27.5)	*p* = 0.581
Hypoperfusion score (%)	47 (41–59)*	57 (47–68)	*p* = 0.009
Proximal occlusion	14 (29%)	5 (11%)	*p* = 0.040
Peripheral scars	44 (92%)	42 (93%)	*p* = 1.000
Hypertrophied bronchial arteries	44 (94%)	39 (89%)	*p* = 0.476
Hypertrophied intercostal arteries	28 (60%)	32 (73%)	*p* = 0.268
Hypertrophied phrenic arteries	25 (53%)	32 (73%)	*p* = 0.082
RV hypertrophy	15 (31%)	36 (80%)	*p* < 0.001
Septum bowing	2 (4%)	26 (58%)	*p* < 0.001
RV/LV ratio	0.99 (0.86–1.1)	1.3 (1.1–1.61)	*p* < 0.001
PA diameter (mm)	29 (26–33)	34 (31–36)	*p* < 0.001
PA/aorta ratio	0.91 (0.84–1)	1.09 (1–1.16)	*p* < 0.001

*RV* right ventricle, *LV* left ventricle, *PA* pulmonary artery

*Only 40 patients with CTEPD were evaluated for hypoperfusion score

### Interobserver agreement

Interobserver agreement was excellent for each of the CT scores evaluated.

The Lin’s concordance correlation was very high for the vascular obstruction score (*r* = 0.92; 95% CI 0.87–0.97), the hypoperfusion score (*r* = 0.95; 95% CI 0.92–0.98) and the hyperaemia score (*r* = 0.90; 95% CI 0.84–0.96).

## Discussion

The main result of the present study is the identification of substantial differences in radiological findings of CTEPD and CTEPH patients. Such differences can be clinically helpful to distinguish the two populations. Most importantly, these differences point at a different pathological substrate underlying CTEPD or CTEPH.

Patients with CTEPD showed a vascular obstruction burden which was similar to patients with CTEPH. As a matter of fact, proximal arterial obstruction (in particular of the right pulmonary artery) was more frequently observed in CTEPD patients than in the CTEPH population; to the best of our knowledge, this finding was never described before. Nevertheless, CTEPD patients had a lesser extension of perfusion defects in the iodine map. Peripheral vascular disease is not clearly visible in morphologic CTPA images, but it can be assessed as perfusion defects in iodine maps. In addition, mosaic hyperaemia, which is also an expression of microvascular peripheral disease, was found in a significantly smaller number of patients with CTEPD. Overall, these data strongly suggest that the absence of peripheral disease (i.e. of small vessel remodelling) might be the cause of the absence of pulmonary hypertension in CTEPD as compared to CTEPH patients.

No significant difference was found between the two groups considering the presence of collateral systemic supply; it is known that the presence of collateral systemic supply can help differentiating CTEPH from other forms of PH [16], but in our analysis, this finding was not useful to distinguish CTEPD from CTEPH patients. The hypothesis is that the development of collateral supply is a direct consequence of vascular obstruction, similarly present in both groups as a response to chronic lung ischemia, rather than a consequence of pulmonary hypertension.

Accordingly, no significant difference was found between the two groups in TLCO. In fact, TLCO is overestimated because of back-perfusion of the capillary bed by the extensive bronchial arterial collateral flow. This “luxury perfusion” plays a role in the maintenance of pulmonary parenchymal viability and in carbon monoxide exchange, although it does not improve the oxygen exchange [17, 18].

As expected, the two groups significantly differed substantially considering the indirect signs of pulmonary hypertension, such as an increased PA diameter and a PA/aorta diameter ratio > 1 and RV/LV ratio > 1, which have already been described to correlate with the presence of pulmonary hypertension [19–22].

The main limitation of the present study is that the number of CTEPD patients evaluated is relatively small since it has been enrolled in a single centre and it is likely to represent a selected cohort of patients, as all these patients were referred for pulmonary endarterectomy being highly symptomatic. As a matter of fact, we lack population studies assessing the prevalence and the characteristics of symptomatic and asymptomatic patients with CTEPD and in which characteristics of such patients may differ from those who are referred to expert centres because of disabling symptoms.

We therefore acknowledge that validation in larger (and if possible unselected) series of patients is necessary, standardising the technical approach to the quantification of vascular obstruction and of lung perfusion.

Another limitation is that CT protocols in CTEPD patients are not uniform, but the enrollment period lasted for several years since CTEPD is not a common condition and technical advances occurred during this period.

In conclusion, CTEPD patients show a vascular obstruction burden similar to CTEPH patients, but without CT signs of pulmonary hypertension and mosaic hyperaemia and with a smaller extension of perfusion defects in the iodine map; these findings could be useful to distinguish CTEPD from CTEPH patients. Eventually, identification of CTEPD population with DECT might in the future avoid performing an invasive procedure like right heart catheterisation.

Most importantly, these data suggest that the absence of peripheral microvascular disease, even in presence of an important thrombotic burden, might be the reason for the absence of pulmonary hypertension at rest in CTEPD.

## Abbreviations

CT: Computed tomography
CTEPD: Chronic thromboembolic pulmonary disease
CTEPH: Chronic thromboembolic pulmonary hypertension
CTPA: Computed tomography pulmonary angiography
DSA: Digital subtraction angiography
PEA: Pulmonary endarterectomy
RHC: Right heart catheterisation
TLCO: Transfer factor of the lung for carbon monoxide
